# Recyclability of PET/WPI/PE Multilayer Films by Removal of Whey Protein Isolate-Based Coatings with Enzymatic Detergents

**DOI:** 10.3390/ma9060473

**Published:** 2016-06-14

**Authors:** Patrizia Cinelli, Markus Schmid, Elodie Bugnicourt, Maria Beatrice Coltelli, Andrea Lazzeri

**Affiliations:** 1 National Research Council, Institute for the Chemical and Physical Processes UoS Pisa, Via G. Moruzzi 1, Pisa 56124, Italy; 2 Department of Civil and Industrial Engineering, University of Pisa, Via Diotisalvi 2, Pisa 56122, Italy; mb.coltelli@ing.unipi.it; 3 Fraunhofer Institute for Process Engineering and Packaging IVV, Giggenhauser Strasse 35, Freising 85354, Germany; markus.schmid@ivv.fraunhofer.de; 4 Chair of Food Packaging Technology, Technische Universität München, Weihenstephaner Steig 22, Freising 85354, Germany; 5 Innovació i Recerca Industrial i Sostenible (IRIS), Parc Mediterrani de la Tecnologia, Avda. Carl Friedrich Gauss 11, Castelldefels 08860, Spain; ebugnicourt@iris.cat

**Keywords:** whey protein isolate, enzymatic detergents, recyclability, protease, multilayer films, polyethylene terephthalate (PET), polyethylene (PE)

## Abstract

Multilayer plastic films provide a range of properties, which cannot be obtained from monolayer films but, at present, their recyclability is an open issue and should be improved. Research to date has shown the possibility of using whey protein as a layer material with the property of acting as an excellent barrier against oxygen and moisture, replacing petrochemical non-recyclable materials. The innovative approach of the present research was to achieve the recyclability of the substrate films by separating them, with a simple process compatible with industrial procedures, in order to promote recycling processes leading to obtain high value products that will beneficially impact the packaging and food industries. Hence, polyethyleneterephthalate (PET)/polyethylene (PE) multi-layer film was prepared based on PET coated with a whey protein layer, and then the previous structure was laminated with PE. Whey proteins, constituting the coating, can be degraded by enzymes so that the coating films can be washed off from the plastic substrate layer. Enzyme types, dosage, time, and temperature optima, which are compatible with procedures adopted in industrial waste recycling, were determined for a highly-efficient process. The washing of samples based on PET/whey and PET/whey/PE were efficient when performed with enzymatic detergent containing protease enzymes, as an alternative to conventional detergents used in recycling facilities. Different types of enzymatic detergents tested presented positive results in removing the protein layer from the PET substrate and from the PET/whey/PE multilayer films at room temperature. These results attested to the possibility of organizing the pre-treatment of the whey-based multilayer film by washing with different available commercial enzymatic detergents in order to separate PET and PE, thus allowing a better recycling of the two different polymers. Mechanical properties of the plastic substrate, such as stress at yield, stress and elongation at break, evaluated by tensile testing on films before and after cleaning, were are not significantly affected by washing with enzymatic detergents.

## 1. Introduction

Multilayer films are a combination of two or more thermoplastics co-extruded or laminated to form one homogenous film with distinct individual layers. Multilayer films provide a range of properties which cannot be obtained from monolayer films [1,2]. The best-performing materials are multilayer flexible films that can reach a thickness of 200 µm, in which barrier properties are often provided by ethylene vinyl alcohol (EVOH), a formal copolymer of ethylene and vinyl alcohol, or by polymers such as polyamide [3,4]. The large amount of multilayer systems used in packaging contributes to the load of municipal solid waste.

In several countries the selective collections of urban waste and the further separation of plastics leads to the recycling of homogeneous polymeric fractions in high value applications, such as textile or injection molding. The recycling process allows the recycling of low density polyethylene (LDPE), high density polyethylene (HDPE), polypropylene (PP), and polyethyleneterephthalate (PET) coming from mono-material packages [5,6].

Difficulties occur in the selective separation of these polymers and in the cleaning of the packaging after use affecting the performance of the recycling process. These issues are particularly important in the case of multilayer systems because, to produce them, an adhesive layer is used in between the main polymeric layers, so the different polymeric materials cannot be separated in post-consumer packaging. The case of PET/PE bilayer packaging is quite representative. The bilayer material, coming from films can be recycled only by grinding the multilayer systems and then extrusion of the material. However, PET and PE are immiscible and their compatibility must be improved in order to employ them in high-value applications. This is possible by using proper compatibilizers [7,8,9], but their costs are not worthy of the product obtained. Furthermore, it was observed that the presence of the adhesive, often consisting in poly(ethylene-*co*-vinyl acetate) (EVA), between the two layer can result in yellowing of PET/PE blended granules [10] because of EVA degradation, thus discouraging applications where a light color of the granules is required. Another example is the multilayer packaging containing polyolefins and a barrier layer of EVOH that cannot be separated and is incompatible with PE and PP [11] during the recycling. For this reason, from these multilayer packages, only final recycled products with low mechanical properties can be obtained. The replacement of barrier polymers with other materials, allowing the separation of the different polymeric layers, could be a correct strategy for designing packaging with optimized recycling of the constituent materials.

Protein from different sources have been widely considered for application in film production [12,13,14,15,16,17,18,19,20]. Materials have been developed by application of a whey protein layer to replace currently-used synthetic oxygen barrier layers (ethylene vinyl alcohol, EVOH) with whey protein coatings [21].

Finding a valid commercial use for this protein, which is mainly discarded, currently, and with the aim of increasing the recyclability of the substrate films in order to obtain high-value products, thus increasing the sustainability of the European packaging and food industries is a priority.

Whey is a byproduct of the cheese-making process. It is the liquid resulting from the coagulation of milk and is generated from the cheese manufacture. The technical definition of this material is “those that remain in the milk serum after coagulation of the caseins at pH 4.6 and temperature 20 °C” [22]. From 1 kg of cheese manufacture it is possible to obtain in the range of 9 L of whey.

The most abundant protein forming the whey is β-lactoglobulin (β-Lg) (50%–60%), [23]. The rest of the composition is subdivided in α-lactalbumin (α-La), bovin serum albumin (BSA), immunoglobulins (Ig), and polypeptides proteose peptones (Pp).

Whey proteins, in their native form, are soluble in water, but when denatured they are no longer soluble. The denaturation of the protein depends on the heat load applied and it is possible to split it into two different processes which are unfolding of the proteins, and successive aggregation of the proteins [24]. During the heating, the three-dimensional structure of the proteins is distorted: the molecules unfold and some of the inner structures interact with those of other molecules. This first step may be reversible if the temperature decreases. This means that the structures could return to their original states. During the first heating only non-covalent interactions are involved and, if the proteins are cooled down, they can return to the native conformation [25]. Further heating can induce aggregation; the aggregation is the process after the unfolding and, here, the proteins establish intermolecular bridging reactions into insoluble clumps and, consequently, this process is not reversible [26].

Whey proteins are compact globular proteins that differ both in structure and in properties from each other as result of differences in amino acid composition and sequence [27]. Films of PET coated with a whey protein layer and a multilayer film based on PET coated with whey protein and PE have been developed [28,29].

The cross-linked protein network provides high barrier properties towards oxygen and water vapor. The water vapor transmission rate (WVTR) was in the range of 2–3 g/(m^2^·d) and the oxygen permeability (OP) in the range of 1–2 cm^3^/(m^2^·d bar) (both normalized to a thickness of 100 µm) depending on the formulations. These values confirmed the potential of whey coating to replace synthetic polymers. The transparency, thermal and mechanical properties, and stability of the films were also compatible for food packaging applications. However, the possibility of separating the PET and PE layers after PET/PE disposal by removing the protein based layer should be investigated. It was, thus, important to address their end of life scenario with the purpose of promoting sustainability by the development of a recyclable packaging material *versus* conventional multilayers mostly directed to incineration.

Thus, the possibility of separating the PET and PE layers after PET/PE disposal by removing the protein based layer should be investigated.

In this context, the present work aimed to investigate the feasibility of a simple procedure for the whey removal, replacing the chemical detergents used for washing the plastic film scraps in recycling facilities with enzymatic detergent for their posterior direction to conventional recycling processes developed for chemically-homogeneous polymer fractions.

Recycling facilities make use of detergents in water baths to clean the plastic scraps, which are then separated by difference in density and directed to processing into pellets. The use of enzymatic cleaners, *versus* chemical ones is very important as they are not aggressive and they do not degrade the structure of the polymeric substrate [30]. Studies report that cost-neutral replacement of surfactant with enzymes in a standard detergent allows for a reduction of washing temperatures and environmental impact without compromising the total washing performance [31,32]. The use of enzymes in detergent formulations is now common in developed countries, with over half of all detergents presently available containing enzymes with costs comparable to chemical-based detergents; thus, the detergent industry is the largest single market for enzymes at 25%–30% of total sales [30].

Enzyme-supported separation of the whey protein coating and the synthetic PET and polyolefin films is one important objective for achieving recyclability in multilayer films and replacing conventionally-used PET/PE films in the packaging industry.

The aim of this study was to investigate the possibility of separating the layers of multilayer films by removal of whey protein-based coatings with enzymatic detergents. Respective trials were performed and evaluated where the whey proteins constituting the coating shall be degraded by enzymes so that the coating films can be washed off from the polyolefin layer to allow further individual substrate recycling. Corresponding enzymatic-based detergents, dosage, time, and temperature, which are further applicable to industrial waste recycling, were determined to optimize process efficiency in view of its possible industrial utilization.

## 2. Materials and Methods

### 2.1. Materials

Whey proteins were from BiPro Davisco Food International Inc. (Le Suer, MN, USA). Whey protein coating solutions were applied on different substrates, such as polyethylene terephthalate (PET), using a standard roller coating operation, followed by a drying/curing stage by hot air convection in order to obtain a stable homogeneous whey film on the substrate, as described elsewhere [28,29].

The films were based on commercial PET film respectively PET Melinex 401, DuPont Teijin Films SA (Contern, Luxemburg) with 50 µm thickness used for bi-layer films, and PET Mitsubishi Polyester Film GmbH (Wiesbaden, Germany), with 20 cm width and 12 µm thickness used for multilayer films, and PE film was DEFA-Folien GmbH (Lohmar, Germany) with 20 µm thickness. Adhesive for lamination was Liofol UK 3640/UK 6800 (HENKEL AG and Co. KGaA, Duesseldorf, Germany). Corona pre-treatment allowed substantial adhesion between the substrate and the layer of whey to be achieved [21,28,29].

Enzymatic detergents were Terg-a-zyme, Alcalase, and Savinase. Terg-a-zyme^®^ active powder detergent was from ALCONOX Inc. (White Plains, NY, USA), and is an enzyme-active powdered detergent containing the enzyme proteases, that consist primarily of a homogeneous blend of sodium linear alkylaryl sulfonate, phosphates, carbonates, and protease enzyme.

Alcalase^®^ 2.5 L from CLEA Technologies (Delft, The Netherlands), Type DX is a liquid enzyme preparation containing a protease suitable for use in high temperature, moderate pH, detergent products. Alcalase is produced by submerged fermentation of a selected strain of *bacillus licheniformis*.

Savinase^®^ 16 L is a liquid enzyme preparation, containing a protease suitable for use in low-temperature detergent preparations. Alacalse and Savinase were purchased from Novozyme (Bagsvaerd, Denmark).

Food-grade glycerol and sorbitol by Merck Schuchardt OHG (Hohenbrunn, Germany) were used as plasticizers. Whey proteins isolate (WPI)-based formulations were prepared by heating to 90 °C for 30 min, which is above both the β-lactoglobulin denaturation temperature (75–80 °C), and either the denaturation temperature of α-lactalbumin (70–90 °C) and bovine serum albumin (about 60 °C) [26,33] to denature the proteins and to facilitate the formation of intermolecular disulfide bonds [29,33]. The result of the combination of these intermolecular bonds with the intermolecular interactions among the protein chains (covalent bonding, hydrogen bonding, hydrophobic interactions, and electrostatic forces) leads to brittle films. Plasticizers are added to lower these interactions between the chains and increase the flexibility of the films, thus avoiding brittle fracture [29,34,35,36].

### 2.2. Methods

The PET film was coated with a layer of whey protein based on a 10% by weight solution of whey protein isolate with a 45/100 by weight of whey/glycerol, and based on a 10% by weight solution of whey protein isolate with a 100/100 by weight of whey/sorbitol. Weighing of dry samples without and with the whey layer allowed the estimation of a 16% by weight contribution from the whey layer to the coated film.

Trials of cleaning were performed just by soaking samples of films, respectively, in a 2% by weight solution of Terg-a-zyme in demineralized water, for 24 h at room temperature with no stirring, thus simulating a pre-soaking treatment of the films.

Samples were also tested in a 1% by weight solution of Terg-a-zyme for 1 h or 2 h in presence of mild stirring, simulating a washing process in recycling facilities. Samples were tested in triplicate and values averaged. Similar tests were performed with water without any detergent as a blank reference.

In tests performed with Terg-a-zyme, Savinase, or Alcalase on multilayer films, the stirring speed was set between 400 and 500 rpm.

For water temperature and time of treatment, different combinations of these variables were tested:

Temperature ranges between 25 °C and 50 °C;
Time ranges between 1 h and 4 h.

The solubility in water is defined as the percentage of film dry matter dissolved after a fixed time of immersion in water. The percent total soluble matter was calculated from initial and final dry weights and reported on dry weight basis.

In order to simulate washing of plastic film waste after grinding in recycling facilities, about 500 mg of films strips 0.5 × 2 cm were introduced in 200 mL of a 4% by weight water solution of Terg-a-zyme and kept under magnetic stirring for 1, 2, and 4 h, respectively.

After drying in an oven at 60 °C for 4 h, the samples were mounted on the supports for analysis by Fourier Transmission Infrared (FT-IR) Nicolet T380 FT-IR (Thermo Scientific, Karlsruhe, Germany), with a resolution of 4 cm^−1^. Samples were cut with a diameter of 17 mm, and scanned over the entire area of wave numbers between 4000 and 250 cm^−1^.

The samples (5 mg) were analyzed by a TA500 thermogravimetric analyzer (TA Instruments, Newcastle, DE, USA) by heating from 25 °C to 600 °C at 10 °C/min scanning rate, under nitrogen atmosphere and the residue was evaluated as the residual weight at 600 °C.

After the removal of the whey-protein-based coating, the mechanical properties of the films were evaluated by a tensile test with an Instron 4302 machine (Canton, MA, USA). The specimens were “dog bone” stamps cut from the films. The area for the test, defined by an extensimeter, had a 6 mm width and 30 mm length. The load cell used was 1 kN, and the test speed was 24 mm·min^−1^. All tests were performed at room temperature using at least five specimens for each sample [37]. 

## 3. Results

### 3.1. Bilayer (Coated) Films

Trials of cleaning of PET/whey films performed in water, without any detergent, gave negative results in removing the whey proteins layer, even with a water bath at 50 °C. Traces of whey protein were present on the PET films and, in particular, there was no unfolding of PET and PE films when the multi-layer structure was treated. This behavior was explained considering that, as anticipated in the introduction, the whey proteins (β-lactoglobulin, α-lactalbumin, and serum albumin) are soluble in water in their native form, while in whey protein isolate, when they are heat denatured and concomitantly cross-linked, they become insoluble. Moreover, the high protein density in the layer applied on PET makes the protein difficult to remove if water alone is used, even with warm temperature.

For these reasons, the use of enzymatic detergents was important to meet the requirements of a possible industrial plant for recycling and for achieving the removal of whey protein from the PET substrate.

Results for samples treated with different procedures are reported in Table 1.

In both samples based on whey glycerol and whey sorbitol, the whey was removed both after soaking in the water solution of the enzymatic detergent with no stirring for 24 h, and after washing with mild stirring for a shorter time (1–2 h). The removal of the protein was in complete agreement with previous studies attesting that, for a coating of 17% by weight [21,29] the removal was faster and more efficient in the presence of stirring. The removal of whey/sorbitol was confirmed by FTIR analysis.

**Figure 1:**
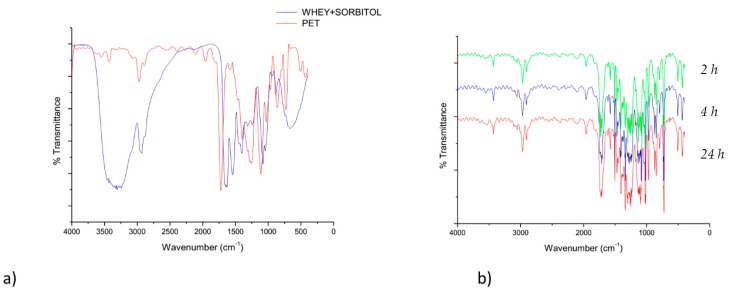
FT-IR spectra of (**a**) PET and whey/sorbitol samples; (**b**) PET/whey sorbitol after washing, respectively, for 2 h, 4 h, and 24 h.

**Figure 2:**
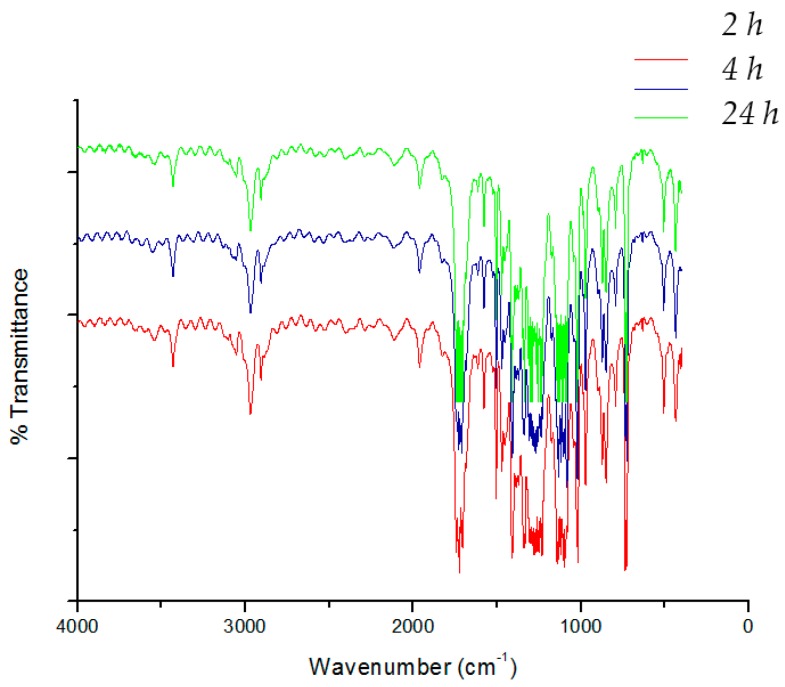
FT-IR spectra of samples with glycerol after washing, respectively, for 2 h, 4 h, and 24 h.

As an example, Figure 1a illustrates the FT-IR spectra of the PET film and a whey/sorbitol cast film, while Figure 1b reports the FT-IR spectra of a whey/sorbitol (PET coated with whey and sorbitol) sample after washing with the enzymatic detergent, Figure 2 reports as well PET coated with whey glycerol after washing.

It is possible to observe that the spectrum of plasticized whey is easily distinguishable from that of PET because of the intense band attributable to both N–H and –OH stretching at about 3450 cm^−1^ and of the typical amide I and amide II band at 1640 and 1560 cm^−1^ due to the C=O stretching and N–H bending, combined with C–N stretching, respectively. The spectrum of PET shows the typical C=O stretching peak at 1720 cm^−1^ and the C–O and C–O–C stretching bands at 1250 and 1100 cm^−1^, respectively. The baseline of the transmission spectra recorded onto washed films shows artifacts named “interference fringes” typical of samples of thin, non-scattering, and of uniform thickness [38]. These artifacts can be easily found in polymeric films produced by flat die extrusion and cannot be eliminated. Despite of the presence of these artifacts, it is evident that the spectra recorded after washing with enzymatic solutions are similar to the spectrum of pure PET and the characteristic peak of the proteins described above are not present in samples treated with enzymatic detergent. This observation suggests that the washing method provided an efficient removal of the whey/sorbitol coating from the PET surface.

The protease enzyme in Terg-a-zyme detergent is bacillus licheniformis subtilisin Carlsberg. Subtilisins are extracellular alkaline serine proteases, which catalyze the hydrolysis of proteins and peptide amides, their activity has been investigated in several conditions [39]. A similar result was obtained by repeating the washing on PET films coated with whey/glycerol, suggesting that the efficiency of the enzymatic treatment is flexible *versus* the type of plasticizer used.

As additional proof of the total removal of the whey applied on the PET substrate, the data of thermal degradation for the whey, whey/sorbitol mixture, and the PET film coated with whey/sorbitol were also compared. Table 2 reports the thermal degradation data for the sample of whey, Table 3 of whey and sorbitol, while Table 4 reports the thermal degradation of PET film coated with whey sorbitol.

The mass loss at 58.7 °C for whey, and 77.5 °C for whey/sorbitol, are attributed to humidity loss. The mass loss at 185.9 °C, reported in Table 3, is attributed to the evaporation of sorbitol; thus, it is not present on raw whey (Table 2). In whey (Table 2) the thermal degradation mass loss at 215.7 °C, and 302.3 °C, which are shifted and merged with those of sorbitol in the whey/sorbitol sample (Table 3), are attributed to the denaturation, and then degradation, of the different protein chains [33] in agreement with degradation reported for similar samples based on milk [40] and whey protein isolate in the presence of polyols [41,42]. 

In the case of PET/whey films, the weight loss recorded for the temperature where the degradation of the protein and sorbitol takes place (14.7%) is similar to the weight loss (16%) obtained in the washings with enzymatic detergent, which is also in agreement with the calculated weight gain due to the layer of whey on PET films, estimated by weighing dry PET films, and dry PET/whey films. Thus, by also considering the results of Table 1, Table 2 and Table 3, the protein/sorbitol layer seems to no longer be present anymore in the washed film. The degradation step at 423.5 °C is attributable to PET thermal degradation [43]. All of the films present a thermal stability compatible for practical application, such as packaging.

### 3.2. Multilayer (Laminated) Films

**Figure 3:**
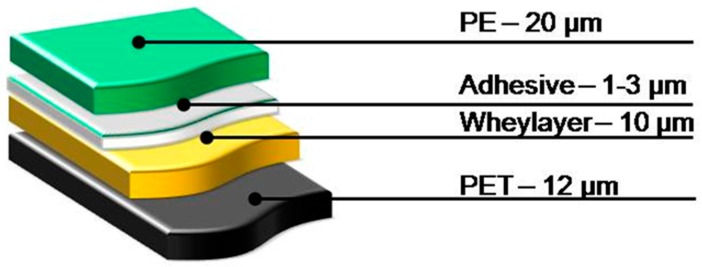
Composition of multilayer film with a whey protein-based barrier layer.

Positive results in producing PET film coated with a whey layer, and the interesting improvements in oxygen barrier properties [21,27,28,29], induced the investigation of multilayers film based on PET, whey layer, and PE. Several compositions of films were investigated and the best performing selected for practical application is reported in Figure 3. For this assembly of films the weight of whey protein present in the dry samples of the film is 17% of the total weight [21,29], as calculated by weighing the same area of the PET, PE films and PET, whey, and PE films.

Results of the whey layer removal, as reported for simulating the process performed in a recycling facility, are summarized in Table 5.

**Figure 4:**
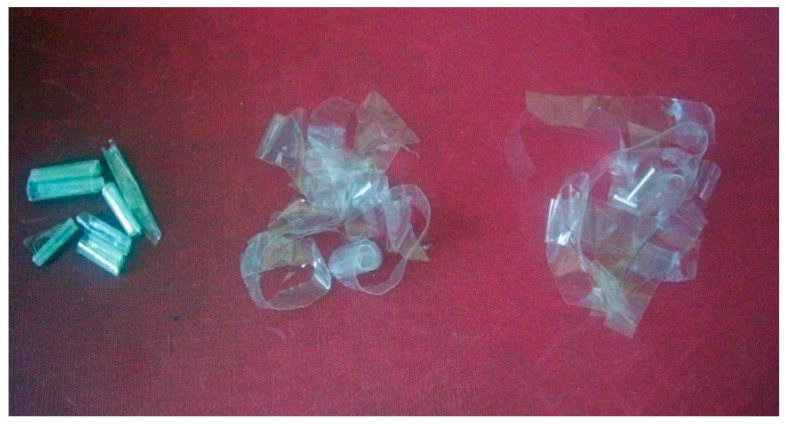
PE/Whey/PET films after treatment with 4% Terg-a-zyme for 1, 2, and 4 h.

Removal of whey layer was efficient after 2 h treatment at room temperature or 1 h at 50 °C, (over 16.5%) and the PE and PET films were separated. Images of films after treatment for 1, 2, and 4 h are reported in Figure 4.

Thus, the Terg-a-zyme was efficient for removal of the whey coating in shredded films and sandwiched with PE and PET. The time of removal of the whey layer and separation of the PE and PET films may be lowered by bathing in warm temperature (50 °C) and with good stirring.

**Figure 5:**
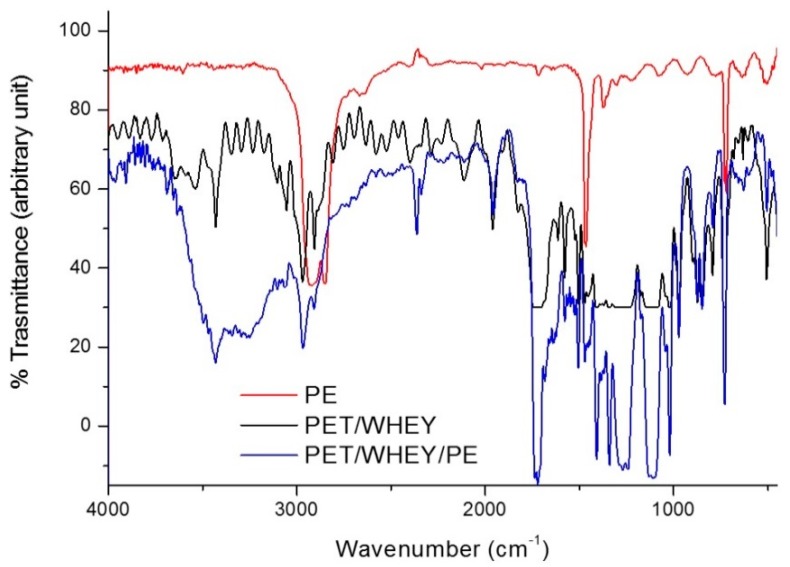
FT-IR spectra of multi-layer samples PE, PET/Whey, and PET/Whey/PE.

In Figure 5 the FT-IR spectra of the multilayer PET/Whey/PE film, and of the PE substrate are reported. In the PE spectrum only the stretching of CH bonds (2910 and 2846 cm^−1^) and bending of CH_2_ (1462 and 720 cm^−1^) are present, whereas in the PET/Whey/PE spectrum the superimposition of bands of whey, PET, and even PE can be noticed.

In order to set a washing procedure which was not dependent just from one type of commercial detergent, other commercial detergents were tested for whey removal; in particular, detergent marketed as Alcalase and Savinase produced by a different company, thus presenting different optimal conditions of operation. Alcalase^®^ 2.5 L, Type DX is a liquid enzyme preparation containing an endo-protease of the serine type suitable for use in high-temperature, moderate pH, detergent products. Alcalase is used in detergent formulations to remove protein-based strains and it has very broad substrate specificity. In other words, it can hydrolyze most peptide bonds within a protein molecule. Peptides are formed which are either dissolved or dispersed in the washing water. Alcalase is active between pH 6.5 and 8.5. It functions between 45 and 65 °C with maximum activity at about 60 °C, above which the activity falls rapidly. Savinase is an endo-protease of the serine type with a very broad substrate specificity. In other words, it can hydrolyze most peptide bonds within a protein molecule. Peptides are formed which are either dissolved or dispersed in the washing water. Savinase functions between 20–60 °C, above which the activity falls rapidly. Its optimal temperature is 55 °C.

The enzymatic additives present in the selected detergent are proteases, which decreases the molecular weight of the proteins, making them soluble and easier to remove from the polymeric matrix. These enzymes improve the cleaning efficiency and operate at low temperature (reducing the energy costs) and in mild conditions. The literature reports the hydrolysis of whey protein by action of different enzymes comprising pepsin, trypsin, and Alcalase [44]. Results of whey removal in multilayer films treated with Savinase are reported in Table 6.

The multilayer disassembles and the removal of the protein layer appears efficient with washing for at least 2 h at room temperature or 1 h at 50 °C.

The optimal performance of these detergents is in warm water (50 °C). However, heating the water bath is an issue for recyclers due to energy consumption with consequent increases in costs, environmental impact, as well as the disposal of warm water. Therefore, the success of the enzymatic treatment at room temperature can boost its applicability. The presence of an enzymatic detergent in the water bath is not an issue for the water disposal since a light organic contamination is already addressed in the system for washing the plastic waste films in particular when these were used for food packaging, which is the main application targeted by these multilayer films [28,29].

In the context of a possible application of the process at a large scale it is interesting to estimate if these detergents can be used more than one time for the removal of the protein layer in the sandwich structure. Thus, tests were performed to evaluate how many times the same detergent (Alcalase at 4% by weight) can be used for the treatment of the multilayer film at 20 °C. Results of weight loss are reported in Table 7.

**Figure 6:**
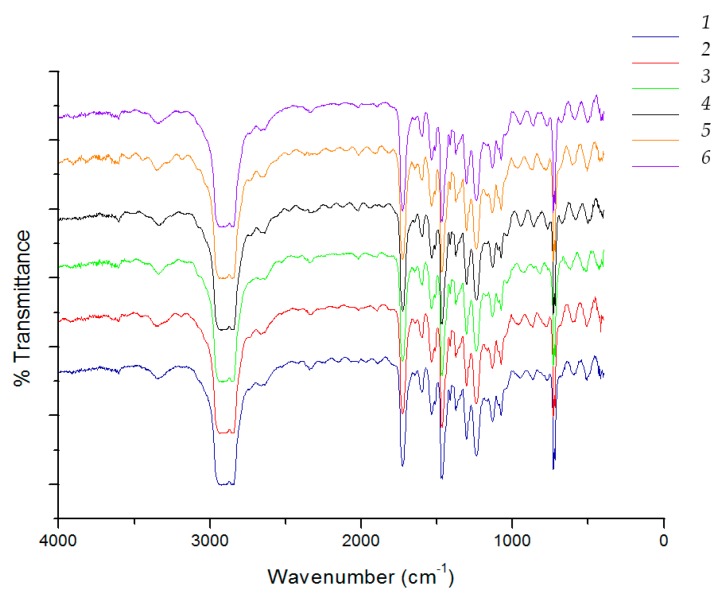
FT-IR spectra of PET in the recovered separated films after washing with Alcalase.

**Figure 7:**
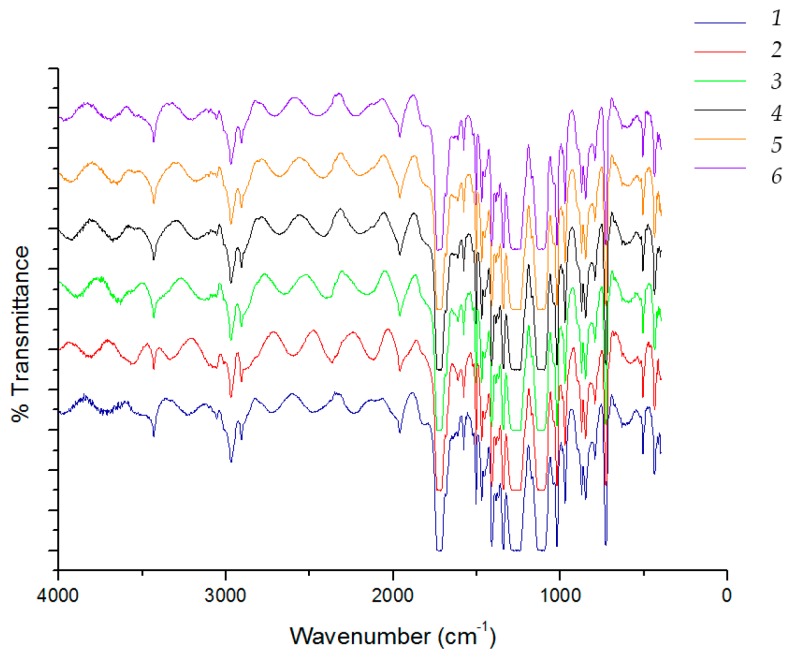
FT-IR spectra of PET in the recovered separated films after washing with Alcalase.

In up to six washings, it was possible to separate the multilayer film and achieve the separation of PET and PE films; for further washing the multilayer was not separated efficiently. The separated films of PET and PE were collected and analyzed by FT-IR spectroscopy. As an example, results on films of PE and PET recovered from the six washings with Alcalase are reported in Figure 6 and Figure 7, respectively. The presence of weak bands at 3400 cm^−1^ due to stretching OH and at 1250 cm^−1^ and 1100 cm^−1^ can be attributable to the presence of residual adhesive deposited on PE film (Figure 5).

In the PET films, the typical bands of whey cannot be revealed, as yet observed in the case of the bilayer system (Figure 1 and Figure 2). The infrared spectra results are, thus, in agreement with a good efficiency of the removal of the whey layer thanks to the enzymatic detergent treatment.

**Figure 8:**
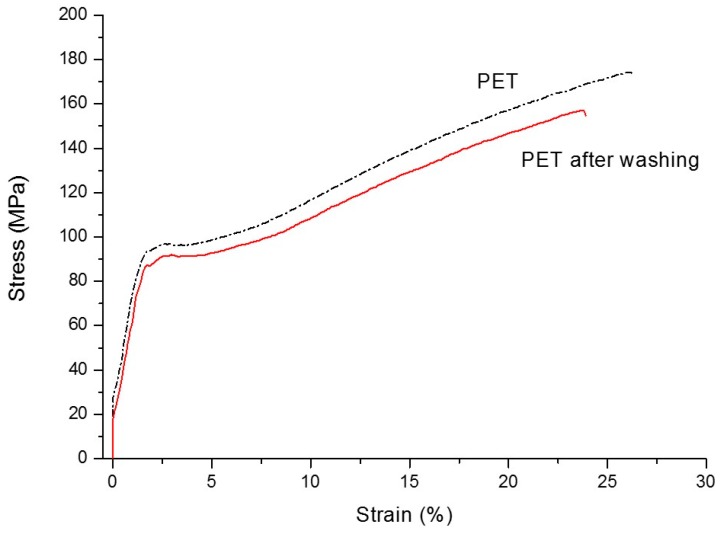
Representative stress/strain curves of PET films before (**left**) and after (**right**) coating with whey and whey removal by washing with Alcalase at 50 °C for 2 h.

Figure 8 shows the behavior during tensile tests of the PET film used for the production of the PET/whey bi-layer, and that of the film recovered after washing with Terg-a-zyme for 2 h at 50 °C. The moderate changes in mechanical properties, related to reduction in the elongation at break from about 28% to 20%, the stress at break from about 160 MPa to 140 MPa, and the lowering in elastic modulus are attributed to the stress underwent by the film during coating and then washing. Elongation and stress at yield are almost unchanged in the two samples. These properties confirm that the removal with enzymatic detergents is not damaging the plastic films whose substrate, PET, is not affected by the action of protease-based detergents, as observed in previous studies [21]. Thus, the use of whey protein confirms to be coherent with a sustainable approach to production of packaging with performing barrier properties and improved recyclability, since the recovered clean fraction could be reprocessed and used in new applications [28,29].

Table 8 reports the mechanical data of PET and PE films, and Figure 8 shows the behavior during tensile tests of the PET film used for the production of PET/whey/PE films, and that of the film recovered after washing with Terg-a-zyme for 2 h at 50 °C. PET film is the most stressed by the production of the multilayer film due to the corona treatment, coating with whey, and then assemble with the PE film. For PET film, the moderate changes in mechanical properties, related to a reduction in elongation at break from about 26% to 19%, stress at break from about 170 MPa to 150 MPa are, thus, attributed to those stresses underwent during production and cleaning. In addition, elongation and stress at yield were almost unchanged in the two samples.

PE film suffers the processing for coupling with PET/Whey, as well as stress during cleaning. These treatments are the same as what PE films would experience during assembling with a PET/EVOH film for production of multilayers, and when disposed and treated in a recycling facility in a water bath with detergents. Thus, the moderate changes in PE properties are attributed to stress in processing, then washing (stirring, and cleaning). These moderate changes in properties confirms that the removal with enzymatic detergents is not damaging the plastic films whose substrates, PET, and coupled film, PE, are not affected by the action of protease enzymes, as observed in previous studies [21]. Thus, the use of whey protein confirms to be coherent with a sustainable approach to production of packaging with performing barrier properties and improved bio-recyclability [45] or recyclability, since the recovered clean and chemically-homogeneous fraction (e.g., PET and PE) could be reprocessed and used into new applications.

## 4. Conclusions

Multilayer films were produced composed of PE and PET films with an interim barrier layer based on whey protein achieving valuable barrier properties. These films were validated for food packaging applications in previous studies and have large potential for being introduced on the market. The washing of samples based on PET/whey and PET/whey/PE was efficient when performed with enzymatic detergent containing protease enzymes. Different types of commercial enzymatic detergents tested presented positive results in removing the protein layer from the PET substrate and from the sandwich films achieving unfolding of the PET and PE films, which allows further separation of the different plastic films by density. These results attested for the possibility of setting the pre-treatment of these multilayer films by washing with enzymatic detergent, selecting the latter among several commercially-available enzymatic detergents.

## Figures and Tables

**Table 1:** Weight loss in PET samples coated with whey and treated with Terg-a-Zyme.

Sample Serial Number	Time-Concentration, Temperature	Temperature (°C)	Weight Loss (%)
Whey/Glycerol	24 h—2% no stirring	20	16.2 ± 0.2
Whey/Glycerol	4 h—1% stirring	50	16.3 ± 0.1
Whey/Glycerol	2 h—1% stirring	50	11.8 ± 0.4
Whey/Sorbitol	24 h—2% no stirring	20	15.8 ± 0.3
Whey/Sorbitol	4 h—1% stirring	50	14.3 ± 0.2
Whey/Sorbitol	2 h—1% stirring	50	14.7 ± 0.4

**Table 2:** Thermal parameter for whey protein isolate.

Tpeak (°C)	Peak Weight Loss (%)	Total Weight Loss (%)	Residue (%)
58.7	5.3	5.3	–
215.7	8.0	13.3	–
302.3	62.7	74.2	24

**Table 3:** Thermal parameter for whey/sorbitol.

Tpeak (°C)	Peak Weight Loss (%)	Total Weight Loss (%)	Residue (%)
77.5	9.7	9.7	–
185.9	17.8	27.5	–
244.0	27.0	54.5	–
303.5	19.4	73.9	–
337.2	15.6	89.5	10.5

**Table 4:** Thermal parameters for sample PET/Whey/Sorbitol.

Tpeak (°C)	Peak Weight Loss (%)	Total Weight Loss (%)	Residue (%)
68.9	0.9	0.9	–
269.1	14.7	15.6	–
423.5	67.7	83.2	–
564.4	1.1	84.3	15.7

**Table 5:** Weight loss in in PE/Whey/PET samples treated with 4% Terg-a-zyme at 20 °C.

Time	Temperature (°C)	Weight Loss (%)
1 h	20	12.3 ± 0.4
2 h	20	16.7 ± 0.3
4 h	20	16.8 ± 0.5
1 h	50	16.5 ± 0.2

**Table 6:** Weight loss in in PE/Whey/PET samples treated with 4% Savinase at 20 °C, and 50 °C.

Time-Concentration	Temperature (°C)	Weight Loss (%)
2 h	20	16.9 ± 0.5
1 h	50	16.8 ± 0.3

**Table 7:** Weight loss in in PE/Whey/PET samples treated with 4% Terg-a-zyme or Alcalase at 20 °C for 2 h.

N Washing with Terg-a-zyme	Weight Loss (%)	N Washing with Alcalase	Weight Loss (%)
1	17.0	1	16.9 ± 0.5
2	16.9	2	17.0 ± 0.3
3	16.2	3	16.8 ± 0.4
4	15.6	4	15.4 ± 0.2
5	15.3	5	15.1 ± 0.3
6	15.4	6	15.3 ± 0.1
7	15.0	7	14.5 ± 0.2

**Table 8:** Stress at yield, tensile strength, and elongation at break of PET film and the same film after the deposition of whey-based coating and successive removal (washing) of it by Terg-a-zyme treatment.

Sample	Stress at Yield (MPa)	Tensile Strength (MPa)	Elongation at Break (%)
PET	98 ± 2	170 ± 20	26 ± 6
PET after washing	98 ± 5	150 ± 20	19 ± 5
PE	12 ± 0.3	27 ± 2	150 ± 12
PE after washing	11 ± 0.1	22 ± 1	280 ± 18

## Abbreviations

The following abbreviations are used in this manuscript:

PET	Poly ethylene terephtalate
PE	Polyethylene
FT-IR	Fourier transform infrared spectroscopy
